# Use of Phenobarbital to Treat Neonatal Abstinence Syndrome From Exposure to Single vs. Multiple Substances

**DOI:** 10.3389/fped.2021.752854

**Published:** 2022-01-31

**Authors:** Alla Kushnir, Cynthia Garretson, Maheswari Mariappan, Gary Stahl

**Affiliations:** ^1^Division of Neonatology, Department of Pediatric, Cooper University Hospital, Camden, NJ, United States; ^2^Department of Pediatric, Cooper University Hospital, Cooper Medical School of Rowan University, Camden, NJ, United States; ^3^Ambulatory Clinical Practice, Cooper University Hospital, Cherry Hill, NJ, United States

**Keywords:** neonatal abstinence syndrome (NAS), opioids, phenobarbital, outcomes, multi-substance use, length of treatment

## Abstract

Drug use in pregnancy is a major public health issue. Intrauterine exposure to opioids alone or in addition to other substances may lead to neonatal abstinence syndrome (NAS). Little consensus exists on optimal therapy, especially for those exposed to multiple drugs. We aim to determine whether the use of opioids alone vs. in combination with phenobarbital will affect short-term neonatal outcomes. This retrospective review of infants admitted to the neonatal intensive care unit (NICU) included newborns ≥35 weeks of gestation exposed to opioids, or multiple substances including opioids, *in utero*. Treatment with opioids alone, and addition of phenobarbital as initial therapy vs. rescue, was evaluated. Out of 182 newborns, 54 (30%) were exposed to methadone alone vs. 128 (70%) to multiple drugs. Length of stay (LOS) in the hospital was not significantly affected (*p* = 0.684) by single vs. multiple drug exposure *in utero*. Treatment of NAS with opioid alone resulted in significantly shorter LOS (27 days), as compared to those treated with opioid and phenobarbital (45 days, *p* < 0.001). LOS was further prolonged in those treated with phenobarbital as a “rescue” medication in addition to an opioid (49 days, *p* < 0.0001). There was a significant increase in LOS and duration of opioid treatment for all infants treated with phenobarbital, both in those exposed to opioids alone, and to multiple substances *in utero*.

## Introduction

The prevalence of opioid use disorder (OUD) has increased substantially over the last decade (1, 2). The drug poisoning death rate has tripled since 1999, and the percentage of heroin-related deaths increased from 8 to 25% from 2010 to 2015 (3). Among pregnant women, the rate of opioid use increased nearly five-fold between 2000 and 2009 (4). The consequences of OUD are especially severe for pregnant women and their infants, often leading to substantial adverse medical, psychological, and social impacts (5).

Neonatal opioid withdrawal syndrome (NOWS) or neonatal abstinence syndrome (NAS) is a multisystem disorder that affects the central nervous system, as well the autonomic nervous system, and the gastrointestinal tract. NOWS is used when discussing patients exposed to opioids only, while NAS is used to describe withdrawal from opioids as well as other substances. NAS is a result of the abrupt discontinuation of the substances used or abused by the mother during pregnancy and may result from multiple drugs which include, but are not limited to, morphine, heroin, methadone, buprenorphine, prescription opioid analgesics, antidepressants, and anxiolytics (6, 7). The withdrawal signs are seen in about 40–80% of the neonates exposed to opioids *in utero* (8).

Early clinical manifestations of NAS are tremors, excessive crying, irritability, and diarrhea. Jitteriness, temperature instability, sneezing, sweating, and mottling are also seen with NAS. Gastrointestinal signs including diarrhea, poor feeding, vomiting, and poor weight gain are also associated with withdrawal signs. Seizures are seen in about 2–11% of the infants with NAS (8, 9). The severity of NAS, onset, and duration of symptoms are all dependent on the type of substances, their half-life, amounts, placental transfer abilities, time of last dose, and other properties of the substances used/abused by the mother.

The management of these neonates begins by using a scoring system for the assessment of the neonates' withdrawal signs. There are multiple scoring systems, but the Finnegan Scoring System is used most commonly to assess NOWS or NAS. Finnegan Scoring System (FSS) or Modified Finnegan Scoring System (MFSS) is a scale that quantifies the severity of the withdrawal signs in NAS of term neonates (10–12). The individual symptoms are weighted depending on the symptom. It is the most comprehensive of the scales used to monitor NAS. (13). showed that the scores of ≥8 should raise concern for withdrawal symptoms and that medical treatment should be initiated at that point (14). Hence, a score of higher than 8 in the Finnegan or Modified Finnegan scoring system is considered clinically significant for withdrawal in neonates. FSS has been documented to have good reliability (15–17). There are multiple other scoring systems, including Eat Sleep Console (ESC) (18–20), which has been extensively used over the last few years.

NAS management is initiated with non-pharmacological care, which includes gentle handling, rocking, feeding on demand, avoidance of waking the sleeping infant, swaddling, and minimum stimulation (16, 21). Pharmacologic treatment is required when the infant fails to respond to supportive care, when the scores remain high, if seizures are seen, or if withdrawal signs are severe as to result in dehydration. Medications used for the treatment of NAS include, but are not limited to, benzodiazepines, buprenorphine, clonidine, methadone, morphine, and phenobarbital (15, 22–27). Information regarding the use of these drugs is summarized by Hudak in the 2012 AAP statement (28). Opioid antagonists, such as naloxone, are contraindicated because they may precipitate seizures in neonates (6), and sedatives such as diazepam and chlorepromazine are not useful due to their prolonged half-life and associated complications (29, 30).

The adverse effect profile of opioids has stimulated further research into the use of other agents, including benzodiazepines, barbiturates, naloxone, chlorpromazine, and clonidine (7, 21, 23, 24, 29–32). A Cochrane review of sedatives for NAS treatment recommends opioids as the initial therapy and phenobarbital as the preferred sedative if a sedative is used (29, 30). The review also proposes that the addition of phenobarbital and clonidine to an opioid may reduce the severity of withdrawal signs and symptoms. However, reviewers advise that more studies are needed to evaluate the safety and efficacy of clonidine and phenobarbital. Currently, there is no consensus regarding which treatment regimen is most effective with the smallest side effect profile.

Phenobarbital has been the drug of choice for sedative-hypnotic withdrawal and had been used as adjunct therapy for NAS due to opioid withdrawal (33). The sedative activity of phenobarbital may be beneficial, but it has little effect on amelioration of the specific opioid related withdrawal symptoms, such as diarrhea and poor feeding (33). The limitation of phenobarbital use include over-sedation, prolonged half-life (45–100 h in neonates), rapid development of tolerance to sedative effects, and high alcohol content of 15% (33).

Our aim was to determine whether the use of opioids alone vs. in combination with phenobarbital would affect length of stay (LOS) and length of treatment of babies exposed to multiple substances *in utero* compared to those exposed to opioids alone.

## Materials and Methods

### Study Methods

A retrospective data analysis of charts of infants admitted to the neonatal intensive care unit (NICU) or transitional nursery in a tertiary hospital in New Jersey from 2007 to 2011 was conducted. Patients included were ≥35 weeks gestational age (GA), whose mothers admitted to using illicit substances or were on Methadone while pregnant, or who had a positive urine drug screen during pregnancy or on admission. Specific drugs, that mothers acknowledge to using and that were found in maternal or neonatal toxicology screen, were documented. Infants with major congenital anomalies and premature infants younger than 35 weeks of GA were excluded.

Neonates who were exposed to multiple substances *in utero* and had signs of NAS for which they were started on an opioid pharmacological treatment were compared to neonates who were started on an opioid in conjunction to phenobarbital. Phenobarbital use was divided into three categories. First category was “No phenobarbital,” where only opioid treatment was used as pharmacological therapy for NAS. Second category of “Phenobarbital and Opioid” was defined as use of phenobarbital and opioid to initiate treatment for NAS, and third category was “Phenobarbital rescue” and defined as use of phenobarbital as an adjuvant therapy due to failure of initial opioid treatment. Opioid treatment failure was generally considered after 3–4 increases in opioid dose; however, that was at the discretion of the neonatal provider.

### NAS Treatment

During the period of data collection there was no standardized protocol in place for the treatment of NAS at our institution. Mothers were encouraged to breastfeed in the absence of illicit substances noted in maternal urine drug screen prior to delivery, as part of the non-pharmacological treatment. All neonates received standard non-pharmacological treatment (such as swaddling, rocking, low stimulation environment, etc.) while being evaluated for signs of withdrawal and NAS. The general practice for most practitioners was to initiate medication when there were three consecutive Finnegan scores 8 or greater. Finnegan scoring was performed every 3–4 h after feeds (interval depended on the frequency of eating for each baby), and changed to every 2 h in those who had a Finnegan score >8. Scoring would return to every 3–4 h once three scores below eight were documented. Both opioids and phenobarbital were given orally (PO). Opioid (tincture of opium initially and morphine in the last 3 years of the study) was started at ~0.02–0.04 mg/kg/dose every 3–4 h and increased as needed. Dose of opioids was calculated to adjust considering multiple types of opioid medication was used.

If there were multiple scores >8 during the day, medication would be increased by ~20% of the current dose. When all scores were <8 for 24–48 h, medication would be weaned by 5–10% of the current dose, at the discretion of the practitioner. Opioid treatment was discontinued when the dose was below 0.01–0.02 mg/kg/dose every 3–4 h.

Some practitioners would start all babies on an opioid and phenobarbital, while others would start phenobarbital in addition to opioid only for neonates exposed to polysubstance *in utero*. In cases of “opioid treatment failure,” all providers would start phenobarbital as a secondary medication. Phenobarbital was typically started at 2 mg/kg/dose PO every 12 h, with a range of 2–4 mg/kg/dose used. Patients were not given a loading dose and typically phenobarbital was not weaned prior to discharge. If withdrawal symptoms were being poorly controlled, phenobarbital dose was adjusted for weight gain, or increased by 20% to a maximum of 6 mg/kg/day at the discretion of the provider. Neonates receiving phenobarbital treatment typically were discharged home on the medication, and would be weaned off in an outpatient setting by the pediatrician.

In the years included in this study, clonidine and buprenorphine were infrequently used in the treatment of NAS. At that time, methadone was being used in the treatment of neonatal withdrawal in other institutions; however, it was not used by our hospital.

Secondary factors associated with maternal drug use (such as maternal tobacco and alcohol use, dose of methadone the mother was on, what other drugs she was positive for on UDS or stated she was using) and medication used for NAS were also compared between the two groups. Primary outcomes include duration of treatment (LOT) in the hospital and length of hospital stay. Logistic regression was performed for factors that were independently significant on LOS and LOT as it related to interaction of phenobarbital and LOS/LOT. MiniTab (Minitab version 15.0, State College, PA, USA) was used to perform statistical analysis. Differences were considered significant at *p* ≤ 0.05.

## Results

Of the 182 charts that were investigated, 54 (30%) of the infants were exposed to methadone alone vs. 128 (70%) to multiple drugs (Table 1). There were no differences in maternal demographic factors based on treatment with methadone alone vs. exposure to multiple drugs. Polysubstance use included use of prescribed and non-prescribed opioids (heroin, oxycodone/OxyContin, etc.), phencyclidine, cocaine, benzodiazepine, amphetamines, and THC.

**Table 1:** Demographics of all neonates with neonatal abstinence syndrome, divided into those exposed to methadone alone vs. those exposed to multiple drugs.

	**Maternal drug use**		***p***
	**Methadone alone (*n* = 54)**	**Multiple drugs (*n* = 128)**	
Sex			
Male, *n* (%)	26 (48)	56 (44)	0.6
Mean maternal age, years (SD)	29.2 (5.1)	28.6 (4.9)	0.4
Race, *n* (%)			
Caucasian	39 (72)	104 (83)	0.2
African–American	8 (15)	10 (8)	
Hispanic	7 (13)	11 (9)	
Cesarian section, *n* (%)	17 (31)	38 (29)	0.8
Mean birth weight, grams (SD)	3,000 (522)	2,852 (483)	0.1
Mean gestational age, weeks (SD)	38.5 (1.6)	38.2 (2)	0.2
Maternal tobacco use, *n* (%)	25 (46)	67 (52)	0.5
Maternal alcohol use, *n* (%)	2 (4)	9 (7)	0.4
IUGR, *n* (%)	5 (9)	8 (6)	0.5
SGA at birth, *n* (%)	6 (11)	17 (13)	0.7
Apgar at 5 min, mean (SD)	8.7 (0.8)	8.6 (1.2)	0.6
Seizures, *n* (%)	4 (7)	7 (5)	0.6
Breast fed, *n* (%)	5 (9)	6 (5)	0.3
Hour of life full oral feeds achieved, mean (SD)^*^	3.8 (19.2)	10 (24)	0.3

* *Data from 75 patients available*.

*IUGR, intrauterine growth restriction, <10% of predicted fetal weight for gestational age; SGA, small for gestational age, birth weight <10%*.

There was no significant difference in neonatal variables (Table 1) seen between mothers on methadone alone vs. multi-drug users. Table 2 further describes the variables related to NAS treatment in neonates exposed to methadone alone vs. polysubstance exposure. Neonatal length of stay (LOS) was not significantly affected (*p* = 0.684) by exposure *in utero* to single vs. multiple drugs (Table 2). There were no significant differences in LOS or length of opioid treatment (LOT) between newborns treated for NAS exposed to methadone alone vs. multi-drug exposure (*p* = 0.68 and *p* = 0.52, respectively; Table 2). LOS was 44.5 days for the 89 infants (69%) born to multi-drug-using mothers who received phenobarbital (rescue or started with opioid) vs. 26.3 days for the 39 infants (30%) who were treated with opioid alone (*p* < 0.0001).

**Table 2:** Clinical data of all neonates with neonatal abstinence syndrome, divided into those exposed to methadone alone vs. those exposed to multiple drugs.

	**Methadone alone (*n* = 54)**	**Multiple drugs (*n* = 128)**	***p***
Sex			
Male, *n* (%)	26 (48)	56 (44)	0.6
Mean length of stay, days (SD)	40 (18.4)	39 (18.3)	0.7
Mean length of treatment, days (SD)	32 (19.4)	30 (19)	0.5
Maximum dose of opioid used, mg/kg (SD)	0.054 (0.04)	0.057 (0.04)	0.6
Day of life maximum opioid dose used (SD)	8.8 (6.5)	7.8 (6)	0.3
Day of life opioid treatment started (SD)	4.2 (2.7)	4 (2.8)	0.6
Mean Finnegan Score prior to treatment start (SD)	7.8 (1.7)	8.3 (1.8)	0.4
Highest score prior to treatment (SD)	12.7 (2.1)	12.9 (2.4)	0.7

**Figure 1:**
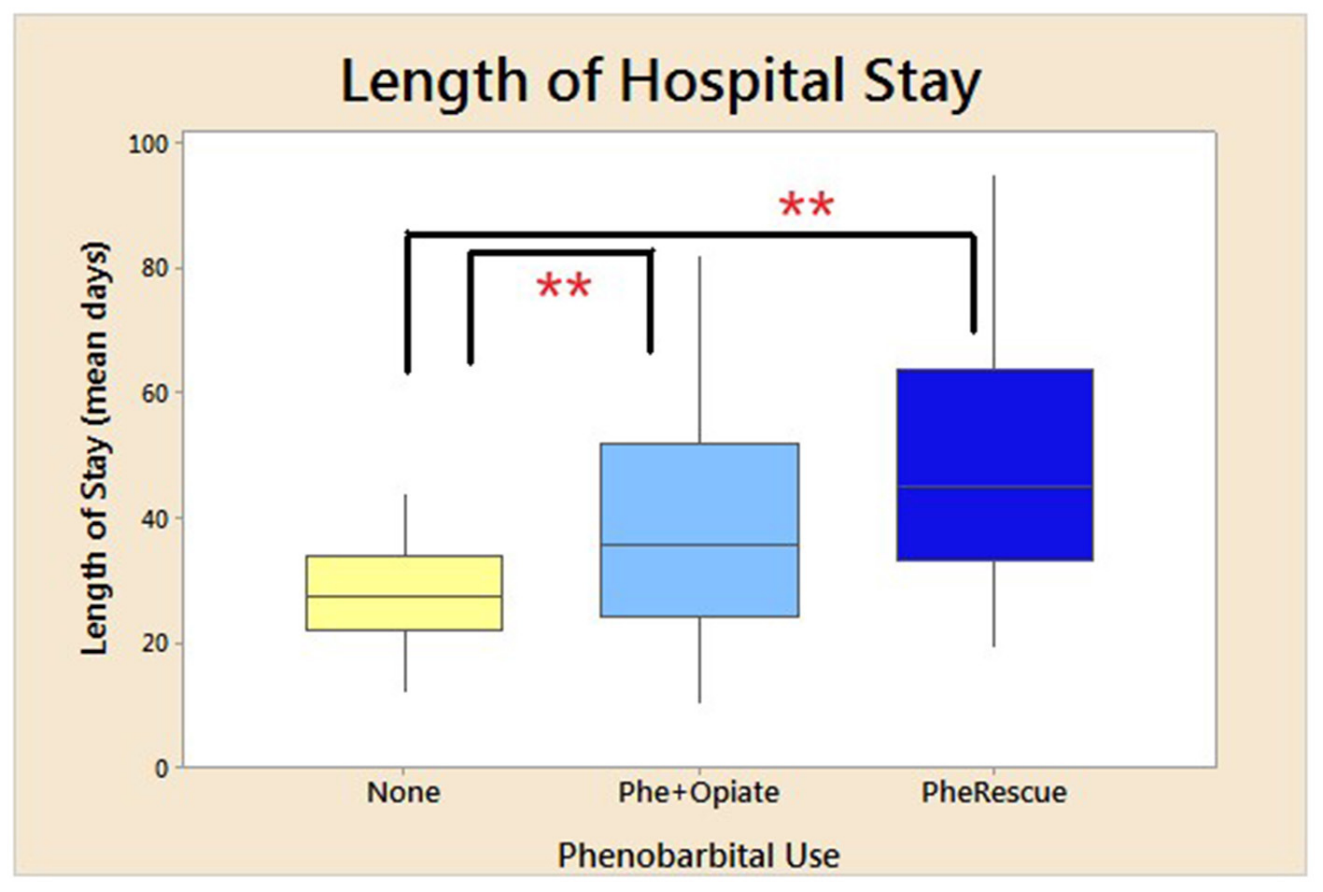
Mean length of hospital stay. Mean duration of LOS for all neonates treated for NAS based on use of phenobarbital. No phenobarbital use and only opioid used for treatment, use of phenobarbital and opioid initiated at the same time, and phenobarbital used as an adjuvant therapy due to failure of initial opioid treatment. *p < 0.05, **p < 0.01. SD for no phenobarbital was 7.5 days; Phe and Opioid, 14.6; and Phe Rescue, 20.5 days.

Table 3 presents demographics divided into neonates treated with phenobarbital and those with no exposure to phenobarbital (Phe). There were no demographic difference in the neonates who were treated with Phe vs. those who were not, except there were more newborns with a diagnosis of seizures at discharge from the NICU in the Phe group (*p* = 0.03; Table 3). Phe was started on day of life 5 for those in the Phe and opioid group and on day of life 14 for those in the Phe rescue group. There was no difference in the severity of NAS in neonates treated with Phe and those who were not (no Phe), as extrapolated from maximum Finnegan scores and mean Finnegan scores the day prior to treatment initiation (Table 4). Maximum NAS score noted during treatment were 13.3 and 12.7 in the no Phe and Phe groups, respectively (*p* = 0.4). There was also no difference in the average 3–5 Finnegan scores documented the day prior to initiation of pharmacological treatment with mean of 8.6 (SD 1.65) and 8 (SD 1.8) in the no Phe and Phe groups, respectively (*p* = 0.25). There was no difference in the rate of treatment with phenobarbital based on exposure to methadone alone or to multiple substances (*p* = 0.9). In the methadone only group, 28, 28, and 44% were in the no Phe, Phe, or Phe rescue groups. While in the poly-substance use group, 30, 29, and 41% were in the no Phe, Phe, and Phe rescue groups, respectively. Table 4 presents data for all neonates with neonatal abstinence syndrome, divided into three groups: Phe and opioid at the same time, Phe as a rescue, and no Phe exposure. Neonates who were not treated with Phe had a LOS of 27, which significantly increased to 38 days in infants who were initiated on phenobarbital and opioid (*p* < 0.0001) and further increased to 49 days for those who received phenobarbital as a rescue medication (*p* < 0.0001; Figure 1; Table 4).

**Table 3:** Demographics of all neonates with neonatal abstinence syndrome, divided into those treated with phenobarbital and those who were not.

	**Phenobarbital treatment**		***p***
	***N*** **=** **182**		
	**Yes**	**No**	
	***n* = 128**	***n* = 54**	
Sex			
Male, *n* (%)	58 (45)	24 (44)	0.9
Mean maternal age, years (SD)	28.9 (5)	28.6 (5.1)	0.7
Race, *n* (%)			
Caucasian	102 (81)	41 (77)	0.7
African–American	11 (9)	7 (13)	
Hispanic	13 (10)	5 (9)	
C-section, *n* (%)	40 (7)	15 (28)	0.6
Mean birth weight, grams (SD)	2,910 (0.5)	2,862 (0.5)	0.6
Mean gestational age, weeks (SD)	38.4 (1.7)	37.9 (2.2)	0.07
Maternal tobacco use, *n* (%)	69 (43)	23 (54)	0.2
Maternal alcohol use, *n* (%)	9 (7)	2 (4)	0.4
IUGR, *n* (%)	10 (8)	3 (6)	0.6
SGA at birth, *n* (%)	17 (13)	6 (11)	0.7
Apgar at 5 min, mean (SD)	8.6 (1.1)	8.6 (0.9)	0.8
Seizures, *n* (%)	11 (9)	0 (0)	0.03
Breast fed, *n* (%)	8 (6)	3 (6)	0.9
Hour of life full oral feeds achieved, mean (SD)^*^	7.4 (22)	10.6 (26)	0.6

* *Data from 75 patients available*.

*IUGR, intrauterine growth restriction; SGA, small for gestational age, birth weight <10%*.

**Table 4:** Clinical characteristics of all neonates with neonatal abstinence syndrome, divided into phenobarbital treatment groups.

	**Phenobarbital treatment**			***p***
	***N*** **=** **182**			
	**Yes**	**Yes, rescue**	**No**	
	***n* = 52**	***n* = 76**	***n* = 54**	
Mean length of stay, days (SD)	38.3 (16.3)	48.6 (19.6)	27.8 (8.7)	0.000
Mean length of treatment, days (SD)	29.7 (17.1)	38.4 (22.2)	20 (8.5)	0.000
Maximum dose of opioid, mg/kg (SD)	0.06 (0.03)	0.06 (0.05)	0.05 (0.03)	0.07
Day of life maximum opioid dose reached, days (SD)	7.7 (5.6)	10.5 (7.5)	5.3 (2.6)	0.000
Day of life opioid treatment started (SD)	5 (3.9)	3.7 (2.3)	3.7 (1.8)	0.02
Mean Finnegan score prior to treatment start (SD)	7.8 (1.9)	8.3 (1.7)	8.6 (1.7)	0.3
Highest score prior to treatment (SD)	12.8 (2.1)	12.6 (1.7)	13.3 (3.4)	0.6

**Figure 2:**
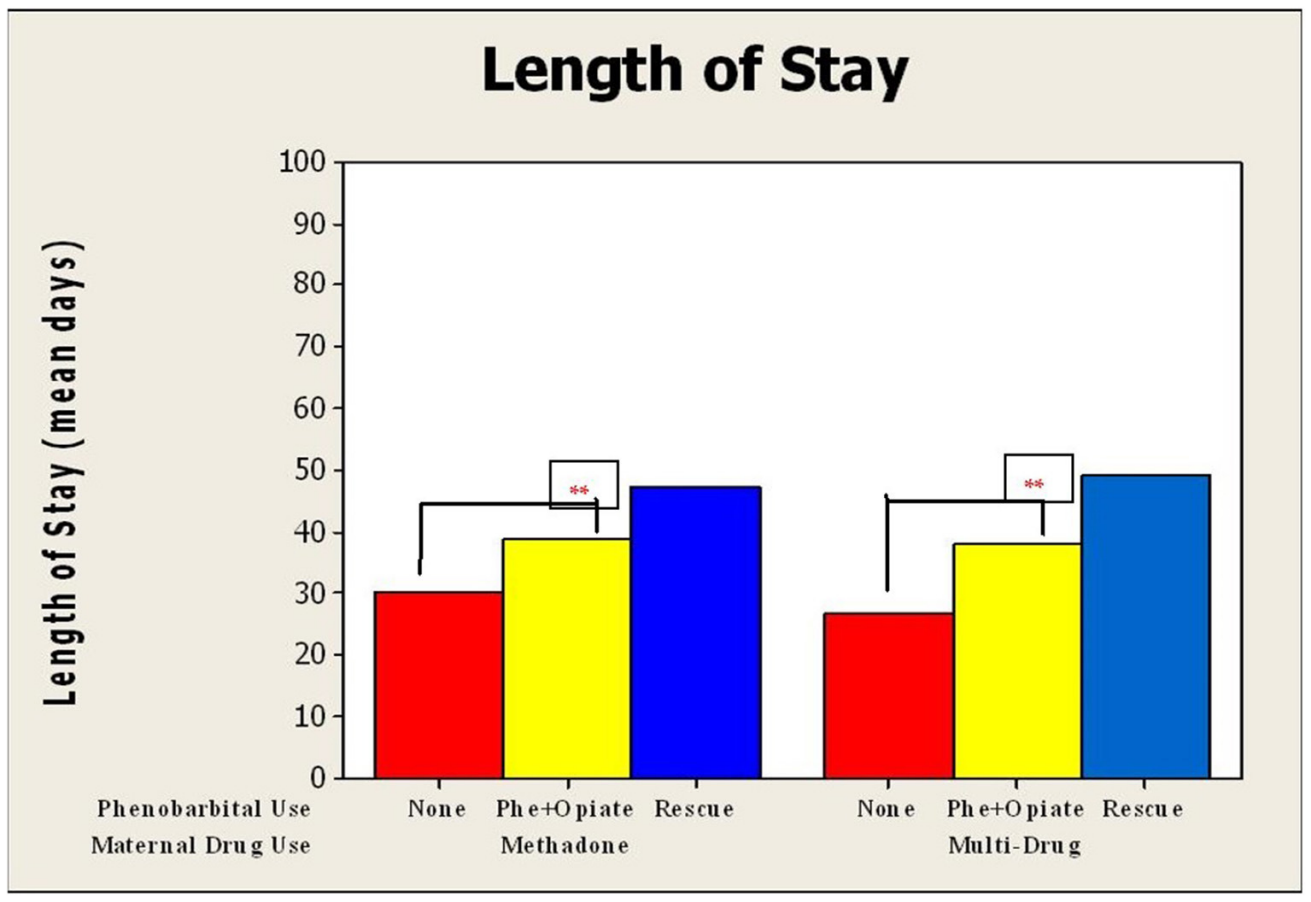
Mean duration of length of hospital stay compared by use of phenobarbital and maternal drug use. Duration of length of stay for neonates who underwent NAS treatment divided into those exposed to methadone alone vs. those exposed to multiple drugs *in utero*. These were further subdivided into no phenobarbital used, phenobarbital, and opioid used from initiation, and phenobarbital used upon opioid treatment failure. For neonates exposed to methadone alone, SD for no phenobarbital was 11 days; Phe and Opioid, 20.6; and Phe Rescue, 18.1 days; and for those exposed to multi-drug, SD for no phenobarbital was 7.5 days; Phe and Opioid, 14.6; and Phe Rescue, 20.5 days. ** *p* < 0.001.

**Figure 3:**
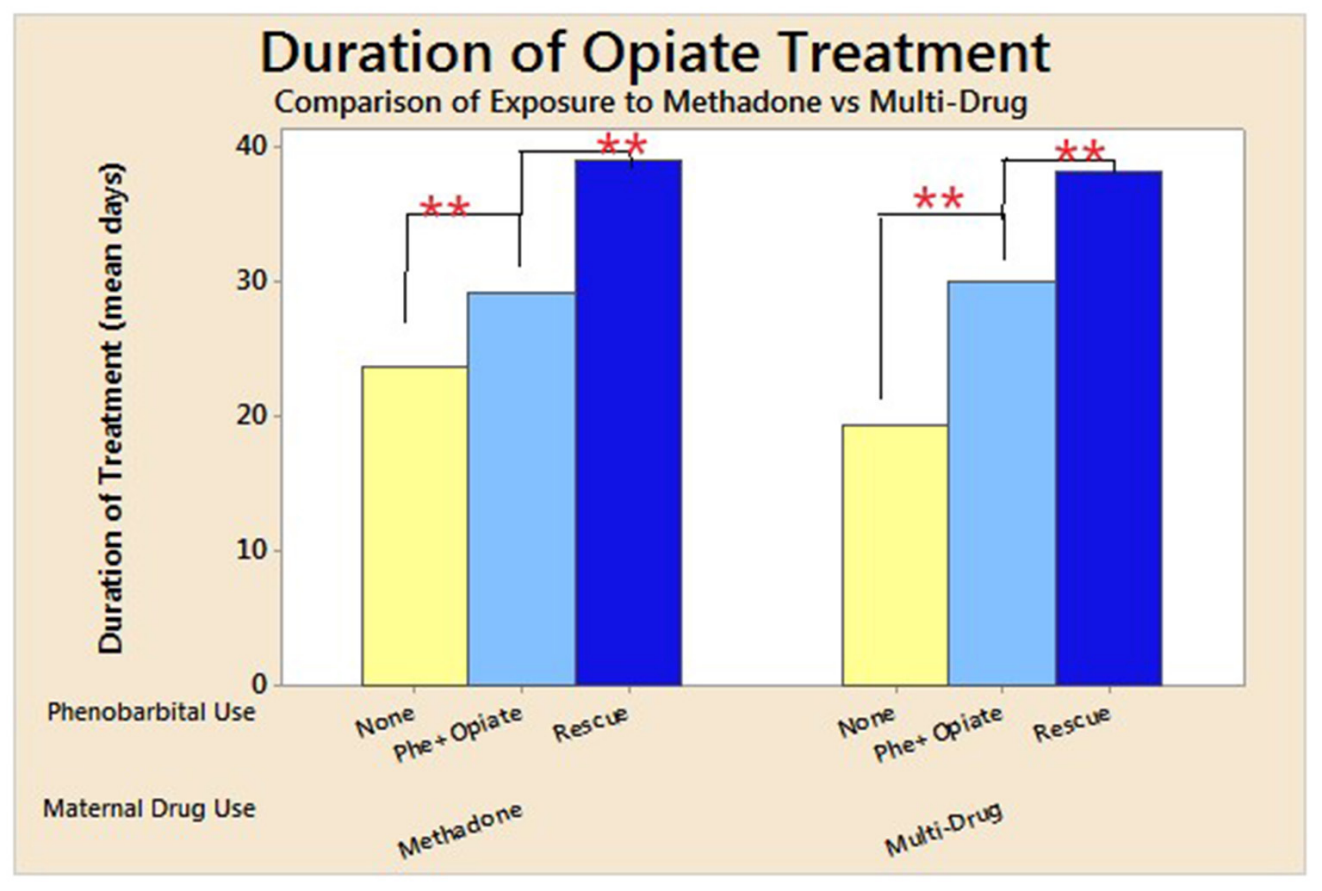
Mean duration of opioid treatment compared by use of phenobarbital and maternal drug use. Duration of length of stay for neonates who underwent NAS treatment divided into those exposed to methadone alone vs. those exposed to multiple drugs *in utero*. These were further subdivided into no phenobarbital used, phenobarbital, and opioid used from initiation, and phenobarbital used upon opioid treatment failure. For neonates exposed to methadone alone, SD for no phenobarbital was 11 days; Phe and Opioid, 20.6; and Phe Rescue, 18.1 days; and for those exposed to multi-drug, SD for no phenobarbital was 7.5 days; Phe and Opioid, 14.6; and Phe Rescue, 20.5 days. ** *p* < 0.001.

There was a significant increase in LOS for neonates exposed to both multiple drugs (*p* < 0.0001) and methadone alone (*p* < 0.0001) *in utero*, who were treated with phenobarbital compared to those who did not receive phenobarbital (Figure 2). Duration of opioid treatment was longer in all babies who received phenobarbital (19.3–40 days, *p* < 0.0001; Figure 3). There was a trend for decrease in the length of stay by 6 days for babies whose mothers received <80 mg of methadone vs. mothers who received >80 mg of methadone (*p* = 0.157).

Maximum opioid dose used during treatment was not affected by use of phenobarbital in either group (*p* = 0.2; Table 4). Phenobarbital continued to be significantly associated with prolonged LOS when a regression analysis was performed to adjust for type of exposure *in utero* (single vs. multiple substances), maternal methadone dose, maximum opioid dose needed to treat neonatal NAS, and day of life maximum opioid treatment dose given to neonate. When adjusted for Finnegan scores prior to initiation of treatment, phenobarbital use trended to prolong LOS (*p* = 0.078) and was associated with significant prolongation in LOS in neonates who received phenobarbital as a rescue medication (*p* = 0.04). There was a significantly longer duration of opioid treatment needed in those who received Phe as a rescue medication (38 days), compared to those treated with Phe and opioid (30 days) or treated with opioid alone (20 days, *p* < 0.0001; Table 4). There was also a significant delay in initiating treatment for NAS and reaching maximum opioid treatment dose in those exposed to Phe (*p* = 0.02 and *p* < 0.0001, respectively; Table 4).

A large portion (89%) of those who received phenobarbital treatment were discharged home on the medication. There was no statistical difference between group 2 (phenobarbital started initially) and group 3 (phenobarbital used as rescue) in the percent of patients discharged home on phenobarbital (*p* = 0.87).

There was no difference in the rate of tobacco exposure in those exposed to methadone alone vs. polysubstance exposure (Table 1), and there was no effect on LOS or LOT based on tobacco exposure (*p* = 0.09 and *p* = 0.14, respectively). Neonates who are having signs of withdrawal consistent with NAS may have difficulty with taking oral feeds (PO). Due to this concern, time to full PO feeds reached between groups was evaluated. There was no difference seen in time to reach PO based on prenatal exposure, methadone alone vs. polysubstance, as seen in Table 1 (*p* = 0.3) or based on treatment with phenobarbital (*p* = 0.6) as seen on Table 4.

## Discussion

NAS has changed extensively over the last 50 years. Today, NAS may be secondary to maternal use of morphine, heroin, methadone, buprenorphine, prescription opioids, antidepressants, anxiolytics, and many other substances. The treatment challenges have worsened with the increase in maternal opioid use, as well as multi-drug use, including prescription and illicit substances (4, 6).

Our study evaluated differences in short-term outcomes, such as length of hospital stay (LOS) and length of treatment for NAS (LOT) based on prenatal exposure to methadone alone vs. multiple substances. A number of studies (34) evaluated the type of prenatal exposure on the effects of neonatal outcomes such as withdrawal symptoms and duration of treatment (25, 35, 36). Unlike the assumption held by many practitioners and results of Janssons' study (34), we did not find increase in LOS or LOT in those exposed to multiple substances *in utero*, as opposed to methadone alone. In the period of the study, Buprenorphine was rarely used to treat maternal addiction in our institution. It is possible that comparing neonates born to mothers treated with Buprenorphine vs. those treated with methadone and/or exposed to multiple substances will show a difference in LOS or LOT. Unlike findings by other studies, including that by Choo et al. (37), we did not find the statistical effect of maternal tobacco use on LOT or LOS.

Phenobarbital (Phe) is a drug of choice for non-opioid NAS (33). Although it had been used as a single therapeutic agent, it is more often used as an adjunct medication to primary opioid therapy (15, 24, 26, 28, 30, 38, 39). Phenobarbital does not prevent seizures at the dosage used for NAS, nor does it improve gastrointestinal symptoms (40). However, it can be used as a second line agent, especially in infants suffering withdrawal from poly-drug exposure (24, 38, 39). While the majority of practitioners use phenobarbital, and it continues to be recommended (28) as a second-line drug if opioid does not control the symptoms adequately (26, 39), it has several known concerns (40). Even though Phe has long been used as an adjunct medication, this study found that using it in babies exposed to multiple substances *in utero* did not improve duration of treatment (LOT) or length of stay (LOS). LOS was prolonged in neonates treated with phenobarbital. Based on studies by Jackson and Ebner, morphine sulfate was preferred to treatment with Phe (35, 41), which is similar to our outcomes. However, neither of those studies differentiated the effect of opioid treatment vs. phenobarbital based on type of prenatal substance exposure.

The results of our study were contrasting to those of Nayeri, which also included newborns exposed to multiple substances, but found no difference in LOS based on treatment with morphine sulfate vs. phenobarbital (25). One of the reasons for this difference is that our institution did not always use a loading dose when initiating phenobarbital, as opposed to Nayeri et al. (25).

Another significant difference in our study compared to prior studies evaluating use of Phe to treat NAS (25, 35, 41, 42) is our use of Phe at initiation of NAS treatment for those neonates with polysubstance exposure or on higher doses of methadone (>80 mg), vs. adding it as an adjunct medication (rescue) after opioid treatment failure. Due to our practice of initiating certain neonates with opioid and Phe, as well as using Phe as a rescue medication after opioid treatment failure for others, we were able to distinguish differences in short-term outcomes based on mode of Phe use. We found that Phe as a rescue prolonged LOS and LOT in neonates exposed to methadone alone, as well as polysubstance exposed neonates.

Due to the retrospective aspect of this study, there may be other reasons for prolonged LOS and LOT in neonates who were treated with Phe. Even though we conducted multivariate analysis to adjust for some of the confounders, it is possible that the babies in the Phe and opioid and Phe rescue groups had “worse withdrawal” or were expected to have “worse withdrawal.” There is a trend for a higher maximum opioid dose and thus may indicate “worse withdrawal.” Based on the 5–10% weaning parameter this can explain a possible 10–12-day difference in LOT. Since day treatment for NAS was started and the day maximum dose of opioid reached were statistically later in the phenobarbital groups (addition or rescue), this may have affected the duration of LOT or LOS. This may be reflected by the multi-use exposure in the groups treated with Phe and the later worsening of the NAS symptoms. This is difficult to determine because it was not seen in the neonates with multiple drug exposure when compared to methadone alone and only when analyzed in relation to exposure to Phe.

There have been several studies evaluating the use of phenobarbital as secondary agent in comparison to clonidine (24, 27, 38). Based on the study by Merhar and Brusseau, patients had better outcomes with phenobarbital as a secondary agent (24, 38); however, Surran et al. (27) noted that clonidine may be as beneficial or more effective. At the time of the study, our NICU did not use clonidine in the treatment of NAS; therefore, we are unable to compare the two treatment modalities.

Even though Phe continues to be recommended as the adjuvant therapy of choice by the AAP (28), there are concerns regarding long-term outcomes after the use of phenobarbital. This is in part due to the high alcohol content of oral phenobarbital as well as its central nervous system depressant qualities, which can lead to significant detrimental effects on neurodevelopment (22). There have been multiple studies to evaluate neurodevelopmental outcomes in infants treated for NAS (43–46); however, they have not evaluated effect of phenobarbital treatment on neurodevelopment in those babies.

The strength of the study was the pragmatic nature of the patient inclusion criteria. Patients were included who were exposed to various substances, including benzodiazepines and nicotine, which have been excluded in some of the previous studies (25).

The limitation of the study was the retrospective nature and, as a result, the inability to randomize the patients. This sample was collected prior to instituting a NAS treatment protocol, and therefore, there were significant variations in practice regarding the initiation of medication(s) and their usage. Due to these variations and the non-standardized manner in which phenobarbital was initiated, it is impossible to determine whether some patients would have had different outcomes without the phenobarbital, or would have needed it at all. Also, due the pragmatic nature of the study, neonates were exposed to various substances other than methadone, including other opioids. This variability may change the NAS treatment need in the babies. Another limitation of the manuscript is that due to the diverse practice habits and due to a lack of protocols, analysis and interpretation of the data was more difficult.

## Conclusion

There was a significant increase in LOS for infants treated with phenobarbital as a rescue compared to those for whom phenobarbital treatment was started in conjunction with an opioid. Overall, use of phenobarbital as a second-line treatment of NAS did not improve the length of treatment of NAS and may have prolonged LOS. Continued research needs to be conducted in evaluation of other medication to be used for neonates exposed to multiple substances *in utero* and those who are not well-controlled with opioid treatment alone.

## Data Availability Statement

The original contributions presented in the study are included in the article/supplementary materials, further inquiries can be directed to the corresponding author/s.

## Ethics Statement

The studies involving human participants were reviewed and approved by the Institutional Review Board of Cooper University Hospital (12-143EX, approved 11/02/2012). Written informed consent from the participants' legal guardian/next of kin was not required to participate in this study in accordance with the national legislation and the institutional requirements.

## Author Contributions

AK and CG: conceptualization. AK, CG, and GS: methodology. AK: formal analysis and supervision. MM and AK: data curation. MM, CG, and AK: writing—original draft preparation. MM, CG, GS, and AK: writing—review and editing. All authors have read and agreed to the published version of the manuscript.

## Conflict of Interest

The authors declare that the research was conducted in the absence of any commercial or financial relationships that could be construed as a potential conflict of interest.

## Publisher's Note

All claims expressed in this article are solely those of the authors and do not necessarily represent those of their affiliated organizations, or those of the publisher, the editors and the reviewers. Any product that may be evaluated in this article, or claim that may be made by its manufacturer, is not guaranteed or endorsed by the publisher.
